# Effect of atomic layer deposition temperature on the performance of top-down ZnO nanowire transistors

**DOI:** 10.1186/1556-276X-9-517

**Published:** 2014-09-21

**Authors:** Suhana M Sultan, Nonofo J Ditshego, Robert Gunn, Peter Ashburn, Harold MH Chong

**Affiliations:** 1 School of Electronics and Computer Science, Nano Research Group, University of Southampton SO17 1BJ, Southampton, UK; 2 Faculty of Electrical Engineering, Universiti Teknologi Malaysia, 81110 Skudai, Johor, Malaysia; 3 Oxford Instrument Plasma Technology BS49 4AP, Bristol, UK

**Keywords:** Zinc oxide nanowire, Top-down fabrication, Field-effect transistor, Atomic layer deposition

## Abstract

This paper studies the effect of atomic layer deposition (ALD) temperature on the performance of top-down ZnO nanowire transistors. Electrical characteristics are presented for 10-μm ZnO nanowire field-effect transistors (FETs) and for deposition temperatures in the range 120°C to 210°C. Well-behaved transistor output characteristics are obtained for all deposition temperatures. It is shown that the maximum field-effect mobility occurs for an ALD temperature of 190°C. This maximum field-effect mobility corresponds with a maximum Hall effect bulk mobility and with a ZnO film that is stoichiometric. The optimized transistors have a field-effect mobility of 10 cm^2^/V.s, which is approximately ten times higher than can typically be achieved in thin-film amorphous silicon transistors. Furthermore, simulations indicate that the drain current and field-effect mobility extraction are limited by the contact resistance. When the effects of contact resistance are de-embedded, a field-effect mobility of 129 cm^2^/V.s is obtained. This excellent result demonstrates the promise of top-down ZnO nanowire technology for a wide variety of applications such as high-performance thin-film electronics, flexible electronics, and biosensing.

## Background

Zinc oxide thin-film transistors are receiving increasing attention because high values of field-effect mobility (3 to 15 cm^2^/Vs) can routinely be achieved in layers deposited at low temperature (<200°C) [1-6]. The values of mobility achievable are significantly higher than those in more well-researched materials such as α-Si/H (approximately 1 cm^2^/V.s), pentacene single crystals (approximately 2.7 cm^2^/V.s), and pentacene thin films (approximately 1.5 cm^2^/V.s) [6]. This makes ZnO-based thin-film transistors very attractive for application in displays, where the higher mobility would provide higher switching speeds or lower power operation. For display applications, ZnO has the additional advantage of high optical transparency, whereas α-Si/H does not. Furthermore, ZnO-based thin-film transistors have considerable potential in emerging applications such as wearable and flexible electronics.

A variety of approaches have been used for the low-temperature deposition of ZnO-based materials, including sputtering [4-6], pulsed laser deposition [7], solution-based processes [8], and atomic layer deposition (ALD) [1-3,9-12]. Of these methods, ALD is particularly attractive because it offers the prospect of an accurate control of material structure in a manufacturing environment. ALD ZnO layers with reasonable electrical and optical properties can be obtained at deposition temperatures below 100°C [13] and even down to room temperature [14,15]. ZnO thin films deposited in ALD exhibit excellent mobility (6 to 30 cm^2^/s) with good stability against stress [10-13]. A good-quality TFT transistor with controlled carrier concentrations was also often obtained with an *I*_ON_/*I*_OFF_ ratio of 10^7^[3,16].

Recently, there has been increasing interest in ZnO-based nanowire transistors fabricated by top-down approaches [17-19] as opposed to the more common bottom-up self-assembly approach [20]. The top-down approach involves material deposition and anisotropic plasma etching to create a nanowire. The advantage of top-down fabrication is that it provides nanowire transistors in well-defined locations on a wafer and enables transistors with different channel lengths to be produced on the same chip. This latter feature is important for the design of practical electronic circuits.

In our previous work [18], we demonstrated a top-down technology that produced transistors with well-behaved electrical characteristics at different channel lengths and with excellent values of breakdown voltage. However, the value of field-effect mobility (0.5 cm^2^/V.s) was at the bottom range of expected values for ALD ZnO thin-film transistors. In this paper, we show how the top-down ZnO nanowire transistor technology can be optimized to give considerably improved values of mobility and drain current. The effects of the ALD deposition temperatures on field-effect mobility are systematically investigated. Nanowire transistor characteristics are compared with the ZnO material properties to determine how the ALD processes influence the transistor electrical characteristics. A field-effect mobility of 10 cm^2^/V.s is obtained at an ALD deposition temperature of 190°C. When the contact resistance is considered, the extracted field-effect mobility of 129 cm^2^/V.s is achieved under *V*_D_ = 1 V.

## Methods

Our top-down ZnO nanowire transistors were fabricated using the technology described in [18], which used an ALD ZnO layer deposited over a SiO_2_ pillar. The ZnO deposition temperature was systematically varied, while all other parameters were kept constant. The atomic layer deposition used 200 cycles of a process comprising an initial Ar purge of 2 s, a 4-s exposure to oxygen plasma, a 1-s (constant) exposure to DEZ, and a final Ar purge of 4 s. The radio-frequency (RF) power and pressure were 100 W and 15 mTorr, respectively. All ALD ZnO films were terminated with the oxygen plasma cycle at the end of each deposition. The thicknesses of the ALD films were measured by ellipsometry and varied somewhat with deposition temperature, from 16 nm at 100°C to 23 nm at 210°C. The ZnO layer was then anisotropically dry etched in an Oxford Instruments Plasma Technology System 100 Inductively Coupled Plasma (ICP) 380 (Oxford Instruments, Yatton, UK) using 25 sccm CHF_3_, 300 W RF power, 1,000 W ICP power, and a pressure of 10 mTorr. The ZnO ICP etch rate was 50 nm/min.

The deposited ZnO layers were characterized using Hall effect measurements of bulk mobility and carrier concentration, sheet resistance measurements for resistivity, and X-ray photoelectron spectroscopy (XPS) measurements of film stoichiometry. The stoichiometry was based on our previous work in [16] which is determined from the ratio of the atomic percentages of the main level Zn-2*p*_3/2_ peak and the O-1*s* binding energy peak observed at 1,022 and 531 eV, respectively. Measurements of nanowire transistor transfer and output characteristics were made on a semiconductor parameter analyzer using the silicon substrate as the back gate. The field-effect mobility was determined from the transconductance using the standard method and the threshold voltage was determined by extrapolation of the linear transfer characteristic. The value of the mobility is determined based on the best performance from each sample.

## Results and discussion

**Figure 1:**
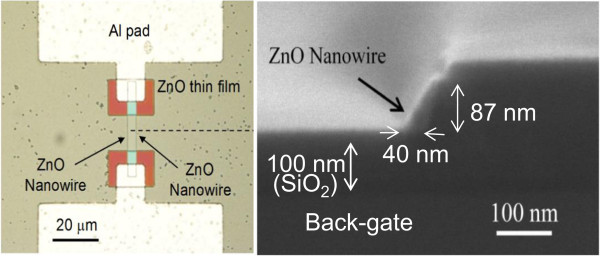
**Fabricated ZnO nanowire FET (left) and cross-sectional SEM image (right) of ZnO nanowire (dotted line).** The ZnO layer was deposited at 190°C comprising an initial Ar purge of 2 s, a 4-s exposure to oxygen plasma, a 1-s exposure to DEZ, and a final Ar purge of 4 s. The ALD RF power was 100 W and the pressure was 15 mTorr.

Figure 1 shows the fabricated ZnO nanowire field-effect transistor (FET) device and a scanning electron microscopy (SEM) cross-sectional image of a ZnO nanowire. The nanowire has a width of 40 nm at the base and a height of 87 nm measured along the pillar. Note that these results were tilt corrected as the sample was titled during the measurement. The nanowire width of 40 nm at the base compares with a thickness of 36 nm measured by ellipsometry after deposition. This is reasonable agreement (11%) given the uncertainties in measuring thickness from an SEM image. The length of the nanowire measured is 10 μm.

**Figure 2:**
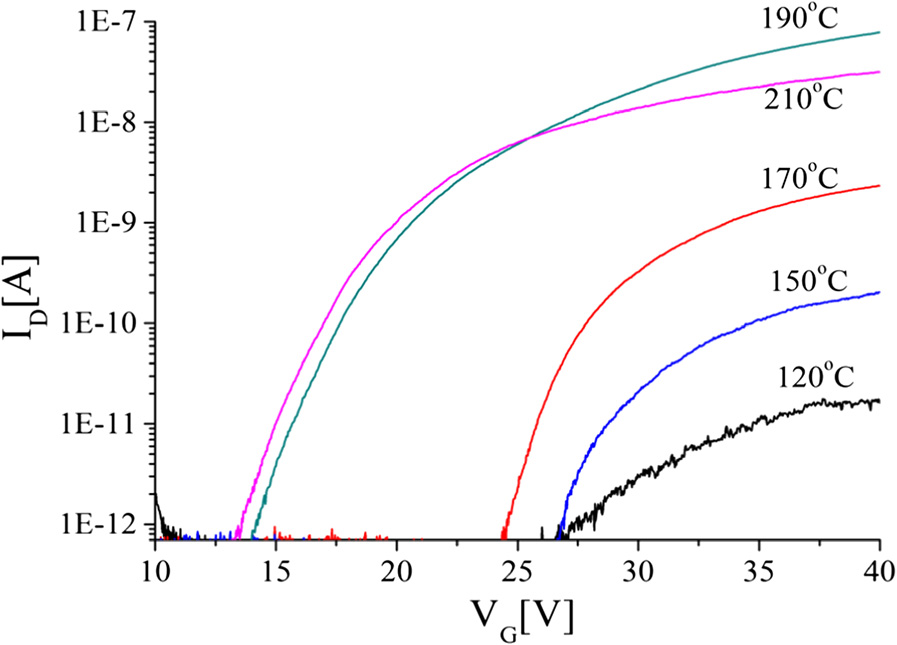
**Sub-threshold characteristics of nanowire transistors with a channel length of 10 μm and two parallel nanowires.** ZnO layers were deposited at different temperatures using an ALD process comprising an initial Ar purge of 2 s, a 4-s oxygen plasma exposure, a 1-s DEZ dose time, an RF power of 100 W, a pressure of 15 mTorr, and a final Ar purge of 4 s. The characteristics were measured at a drain bias of 1 V.

To investigate the effect of ZnO ALD temperature on the nanowire transistors, ZnO depositions were performed at different temperatures in the range 100°C to 210°C, with all other deposition conditions kept constant. Figure 2 shows ZnO nanowire transistor transfer characteristics for devices fabricated on ALD layers deposited at temperatures in the range 120°C to 210°C. The drain current at high gate voltage increases with increasing ALD temperature up to a temperature of 190°C and then decreases at the highest ALD temperature of 210°C. For a gate overdrive, *V*_G_ − *V*_TH_, of 6 V, the drain current increases from 0.014 nA at 120°C to a maximum of 21.1 nA at 190°C and then decreases to 10.5 nA at 210°C. In addition, the sub-threshold parts of the characteristics show a systematic shift towards negative gate voltages with increasing ALD temperature.

Values of threshold voltage were extracted by extrapolation of the linear transfer characteristic and values of field-effect mobility were extracted from the transconductance and are summarized in Table 1. A maximum field-effect mobility of 10 cm^2^/V.s is obtained for an ALD deposition temperature of 190°C. This temperature corresponds with the maximum in the measured drain current. The results in Table 1 indicate that the best value of field-effect mobility is obtained for an ALD temperature of 190°C and that significantly lower values of mobility are obtained at other temperatures.

**Table 1:** Summary of parameters obtained for ZnO nanowire transistors fabricated using ALD layers deposited at different temperatures

**ALD growth temperature (°C)**	**Threshold voltage, *V***_**TH **_**(V)**	**Field-effect mobility (cm**^**2**^**/V.s)**	**Drain current, *I***_**D **_**(nA) at *V***_**D**_ **= 1 V and *V***_**G**_ **−** ***V***_**TH**_ **= 6 V**
120	31	0.1	0.014
150	30	0.6	0.13
170	29	2.5	1.28
190	24	10	21.1
210	22	4.9	10.5

The drain-current was determined from the transfer characteristics at *V*_D_ = 1 V using a gate overdrive (*V*_G_ − *V*_TH_) of 6 V.

**Figure 3:**
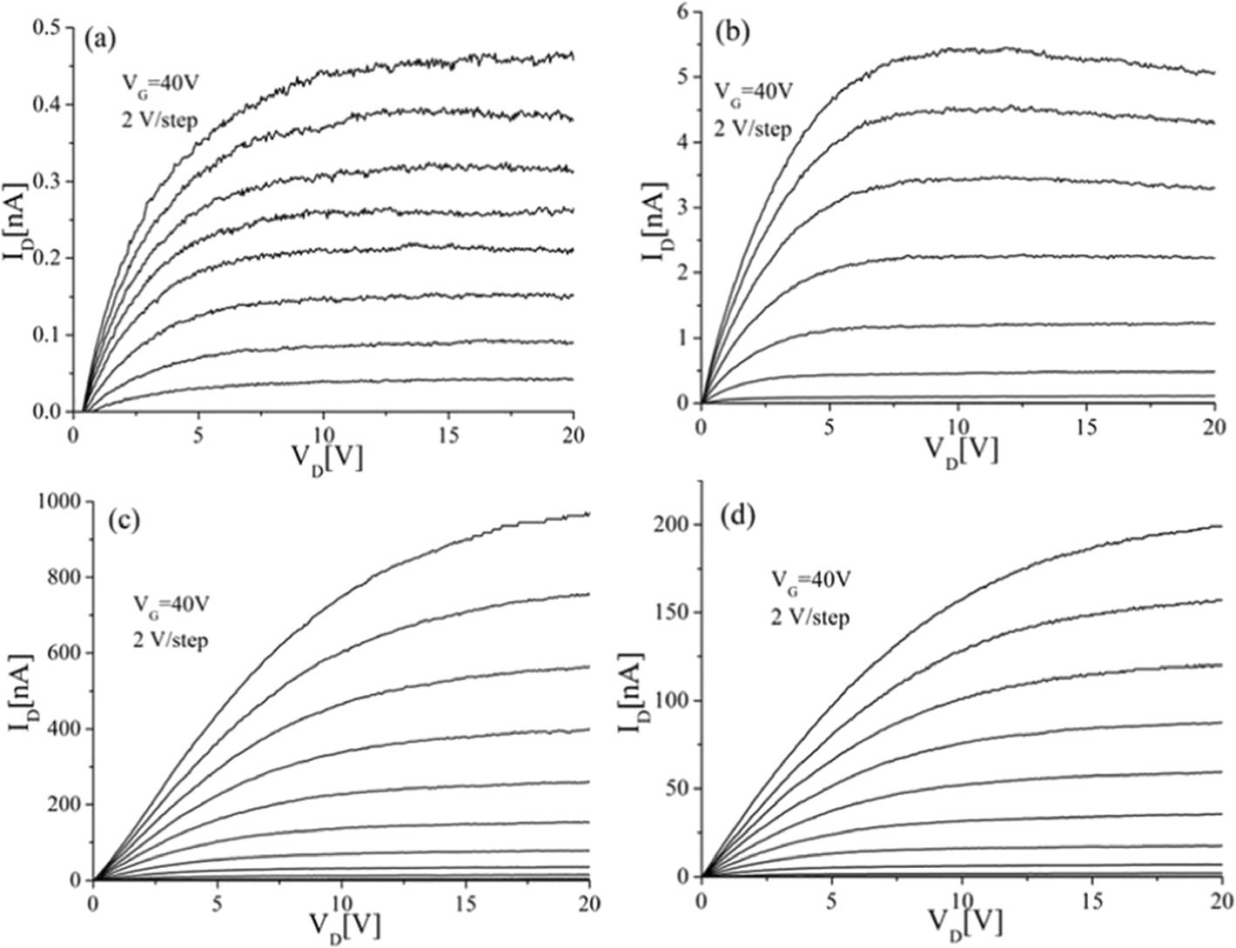
**Output characteristics of nanowire transistors with a channel length of 10 μm and two parallel nanowires.** ZnO layers were deposited at temperatures of **(a)** 150°C, **(b)** 170°C, **(c)** 190°C, and **(d)** 210°C. The ALD process used an initial Ar purge of 2 s, a 4-s oxygen plasma exposure, a 1-s DEZ dose time, an RF power of 100 W, a pressure of 15 mTorr, and a final Ar purge of 4 s.

**Figure 4:**
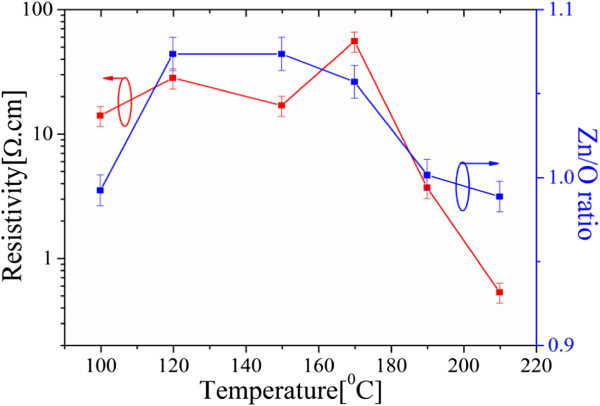
**Resistivity obtained from Hall measurements and Zn/O stoichiometric ratio obtained from XPS.** This ratio is obtained as a function of atomic layer deposition temperature for ZnO layers deposited using an initial Ar purge of 2 s, a 4-s oxygen plasma exposure, a 1-s DEZ dose time, an RF power of 100 W, a pressure of 15 mTorr, and a final Ar purge of 4 s.

**Figure 5:**
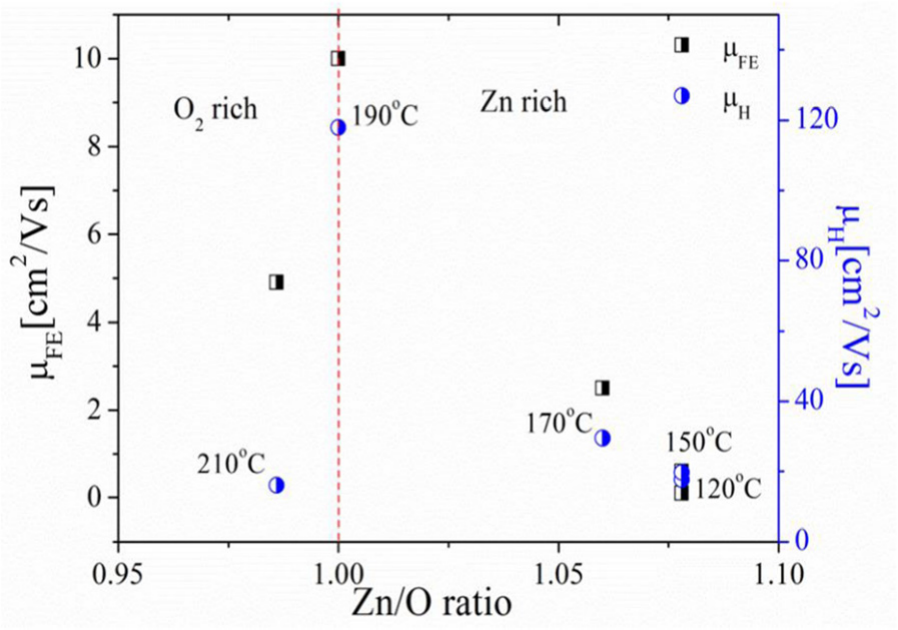
**Field-effect mobility and Hall mobility as a function Zn/O stoichiometric ratio obtained from XPS measurements.** The ZnO layers were deposited using an initial Ar purge of 2 s, a 4-s oxygen plasma exposure, a 1-s diethyl zinc dose time, an RF power of 100 W, a pressure of 15 mTorr, and a final Ar purge of 4 s. The deposition temperature is shown at each measurement point.

Figure 3 shows output characteristics for ZnO nanowire transistors fabricated using ALD ZnO deposited at 150°C, 170°C, 190°C, and 210°C. Well-behaved output characteristics are obtained for all four deposition temperatures. However, it can be seen that the largest drain current is obtained for the 190°C transistor. At a drain bias of 15 V and a gate bias of 40 V, drain currents of 0.45, 5.3, 900, and 187 nA are obtained for ALD temperatures of 150°C, 170°C, 190°C and 210°C, respectively. For a gate overdrive *V*_G_ − *V*_TH_ of 6 V, the values of drain current at *V*_D_ = 15 V are 0.32, 2.8, 150, and 17 nA, respectively. To investigate the origins of the optimum field-effect mobility at 190°C, the ZnO layers were characterized using a variety of techniques.Figure 4 shows the resistivity and Zn/O ratio as a function of deposition temperature. The film resistivity was measured using a four-point probe Hall measurement technique while the Zn/O ratio was measured using XPS. High values of resistivity above 10 Ω.cm are obtained for deposition temperatures up to 170°C, but the resistivity then decreases sharply to 3.4 Ω.cm at 190°C and 0.4 Ω.cm at 210°C. The XPS measurements show that a slightly oxygen-rich film is produced at a deposition temperature of 100°C. At temperatures in the range 120°C to 170°C zinc-rich films are produced while at a temperature of 190°C a stoichiometric film is produced. Finally, at a temperature of 210°C a slightly oxygen-rich film is produced. Results in Figure 4 indicates that at deposition temperature of 190°C, a stoichiometric (1:1) ZnO film and low resistivity value of 3 Ω.cm can be obtained.To further investigate the relationship between film stoichiometry and mobility, Figure 5 shows a graph of field-effect mobility and Hall mobility as a function of film stoichiometry for ALD ZnO layers deposited at different temperatures. A similar trend can be seen for both field-effect mobility and Hall mobility in which the values of mobility fall off sharply as the film composition departs from stoichiometry. While other factors such as surface roughness undoubtedly influence the mobility, this figure clearly demonstrates the importance of ZnO film stoichiometry in determining both field-effect and Hall mobility. The fall-off of mobility is particularly sharp for the oxygen-rich sample that was deposited at 210°C. This sharp fall-off of mobility may be caused by a large surface roughness seen in this sample.The results in Figure 5 also suggest that there is considerable scope for achieving higher values of field-effect and Hall mobility at lower ALD temperatures by adjusting the deposition process to give more stoichiometric films. For example, at temperatures between 120°C and 170°C stoichiometric film could be achieved by decreasing the zinc content in the films.

The Hall effect mobility of 120 cm^2^/V.s obtained in our ZnO thin films is comparable with values in the range 120 to 155 cm^2^/V.s obtained for single-crystal ZnO thin films grown on sapphire substrates using molecular beam epitaxy or pulsed laser deposition [21]. The field-effect mobility of 10 cm^2^/V.s obtained in our ZnO nanowire transistors compares with values of 12.5, 6.7, and 1 cm^2^/V.s reported by Levy et al. [1], Lim et al. [2], and Huby et al., respectively [3], in ZnO thin-film transistors fabricated using atomic layer deposition. ICP etching can therefore be used to produce ZnO nanowire transistors with comparable values of field-effect mobility as obtained in ZnO thin-film transistors, indicating that the ICP etch does not significantly degrade the device performance.

The total resistance, composed of channel and contact resistances, is measured across the nanowire’s (NW’s) output terminals at *V*_G_ = 40 V. The channel resistance in the linear region exhibits a purely ohmic behavior [22] while contact resistance consists of ohmic and a non-ohmic components. The measured total resistances initially reduced from 68.5 GΩ (150°C), 8.5 GΩ (170°C), 111 MΩ (190°C), and finally increased to 531 MΩ for film deposited at 210°C. The doping concentration achieved in our ZnO nanowire transistors is in the range from 1.5 × 10^16^ to 3.0 × 10^16^ cm^−3^ for a deposition at 190°C; so the effect of the source-drain contact resistance (*R*_con_) and channel resistance (*R*_NW_) can limit the drain current and extraction of the field-effect mobility.

**Figure 6:**
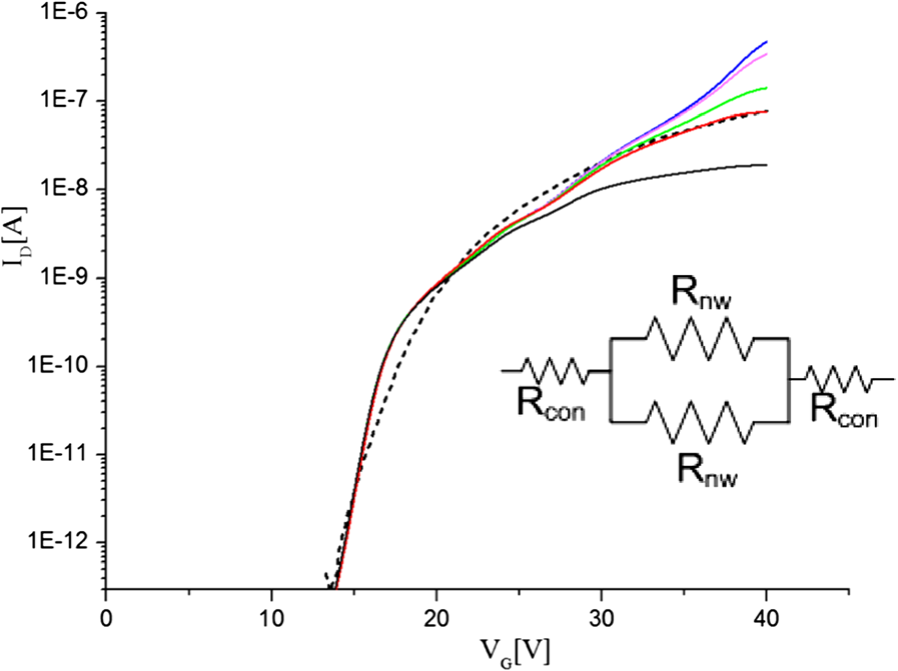
**Simulated sub-threshold *I***_**D**_**-*V***_**G **_**characteristics of ZnO dual nanowire FETs with different values of contact resistance, *R***_**con**_**.** A measured characteristic is shown for comparison for a ZnO layer deposited at 190°C. *V*_D_ = 1 V. (Measured: black dashed line; simulated: blue line 10 Ω, pink line 800 kΩ, green line 5 MΩ, red line 11.4 MΩ, black line 50 MΩ).

To investigate the role of contact resistance, we performed 3D Silvaco Atlas simulations based on the 10-μm, dual nanowire ZnO FET deposited at 190°C. The simulations assumed single-crystal ZnO and used a Shockley-Read-Hall (SRH) recombination model. An SRH model is used in Silvaco to model the density of states with fixed minority carrier lifetimes. In the ZnO nanowire FET, the model focuses on the majority carrier in the channel without the effect of recombination with the minority carriers. The density of states parameters used were based on [23] and [24]. The sub-threshold slope of the measured ZnO nanowire transistor was first fitted by introducing gate oxide interface states into the simulations, following the approach in [25]. Contact resistance was then included in the simulations by modeling the contacts as ohmic with a fixed resistance. Figure 6 shows simulated sub-threshold characteristics for values of contact resistance in the range 10 Ω to 50 MΩ and, for comparison, a measured characteristic. The inset shows the schematic diagram of the resistances in the fabricated nanowire device. The simulations give a drain current *I*_D_ of 0.45 μA (at *V*_G_ = 40 V) when *R*_con_ is 10 Ω, which degrades to 0.015 μA when *R*_con_ is 50 MΩ. Good agreement between simulated and measured nanowire device characteristics is obtained when the contact resistance, *R*_con_, is 11.4 MΩ. Since the total resistance measured for 190°C deposited device was 111 MΩ, 2*R*_con_ of 22.8 MΩ (at the source and drain) is accounted for 20.5% of the total resistance. This has an effect on the drain current and field-effect mobility extraction. The difference in the resistance after removing the contact resistance will be the nanowire resistance which in this case is high due to depletion issues in the nanowire channel itself. This will be investigated in the future.

**Figure 7:**
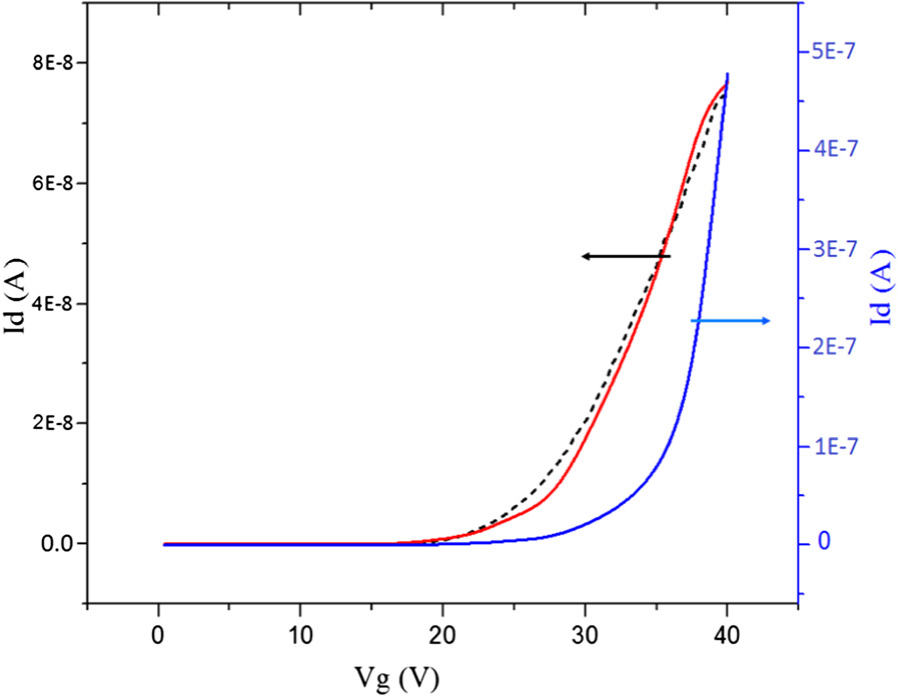
**Simulated linear output characteristic of a dual nanowire ZnO FET.** With a contact resistance of 11.4 MΩ (red curve) and, for comparison, a measured characteristic for a ZnO layer deposited at 190**°**C (dashed black curve). *V*_D_ = 1 V. The blue curve shows a simulated characteristic for a contact resistance of 10 Ω for de-embedding the value of field-effect mobility.

To determine the effect of contact resistance only on the extraction of field-effect mobility, the simulated and measured linear *I*_D_-*V*_G_ characteristics were used as shown in Figure 7. From the differential *∂I*_D_/*∂V*_G_ of the simulated characteristic, a maximum transconductance (*g*_m_) of 8.5 nS is obtained and a field-effect mobility of 8 cm^2^/V.s. These values are in good agreement with the measured transconductance *g*_m_ of 10.4 nS and the measured field-effect mobility of 10 cm^2^/V.s. The effect of contact resistance on field-effect mobility can be de-embedded using the simulated characteristic for a device with a 10-Ω contact resistance in Figure 7. Using the same approach, a de-embedded value of field-effect mobility of 129 cm^2^/V.s is obtained. This value is significantly higher than the measured value of 10 cm^2^/V.s and demonstrates the potential for further improvements in the on-current through modifications to the contact resistance technology.

Considerable research has been published in the literature on bottom-up ZnO NW FETs [26-33], with widely varying values of field-effect mobility. Extremely high mobility values (>1000 cm^2^/V.s) have been reported in passivated ZnO nanowire transistors [26,27] but much lower values (75 to 80 cm^2^/V.s) in unpassivated devices. The results presented in this work were obtained on unpassivated ZnO nanowire transistors. There may also be a scope to further increase the mobility in our devices by using surface passivation.

## Conclusions

This paper has studied the effect of the atomic layer deposition temperature on the performance of top-down, ZnO nanowire field-effect transistors. The ZnO deposition temperature has been systematically varied, with all other deposition conditions kept constant. A deposition temperature of 190°C gives the maximum field-effect mobility of 10 cm^2^/V.s and also corresponds with the maximum Hall effect mobility of 120 cm^2^/V.s. This result is explained by the good stoichiometry of the ZnO films at a deposition temperature of 190°C. The optimized field-effect mobility of 10 cm^2^/V.s is approximately ten times higher than can typically be achieved with thin-film amorphous silicon transistors. Furthermore, device simulations show that the field-effect mobility is limited by contact resistance and when this is de-embedded, the field-effect mobility increases to 129 cm^2^/V.s. It is clear therefore that top-down fabricated ZnO nanowire transistors show considerable potential for high-performance, transparent, thin-film electronics on either glass or polymer substrates.

## Competing interests

The authors declare that they have no competing interests.

## Authors’ contributions

SMS carried out the study, experiments, and characterizations. NJD performed the simulation study. RG advised on the ALD deposition technique. PA, HMHC, and SMS drafted the manuscript. All authors read and approved the final manuscript.
